# Cytoplasmic poly(A)-binding protein 1 (PABPC1) is a prognostic biomarker to predict survival in nasopharyngeal carcinoma regardless of chemoradiotherapy

**DOI:** 10.1186/s12885-023-10629-4

**Published:** 2023-02-20

**Authors:** Ling Feng, Shengen Xu, Xiaochen Li, Xingwang Sun, Wenbo Long

**Affiliations:** 1 Pathology Department of the First Affiliated Hospital, Southwest Medical University, Sichuan, People’s Republic of China; 2 Department of Otorhinolaryngology-Head and Neck Surgery, the Affiliated Hospital of Southwest Medical University, Sichuan, People’s Republic of China

**Keywords:** Nasopharyngeal carcinoma, Molecular marker, Epstein-Barr virus, Overall survival, Disease-free survival, Induction, Concurrent chemotherapy, Intensity-modulated radiotherapy

## Abstract

**Background:**

Nasopharyngeal carcinoma (NPC), especially the nonkeratinizing type, is a malignant tumor primarily occurring in southern China and Southeast Asia. Chemotherapy (CT) and combined radiotherapy (RT) is used to treat NPC. However, the mortality rate is high in recurrent and metastatic NPC. We developed a molecular marker, analyzed its correlation with clinical characteristics, and assessed the prognostic value among NPC patients with or without chemoradiotherapy.

**Methods:**

A total of 157 NPC patients were included in this study, with 120 undergoing treatment and 37 without treatment. EBER1/2 expression was investigated using in situ hybridization (ISH). Expression of PABPC1, Ki-67, and p53 was detected with immunohistochemistry. The correlations of EBER1/2 and the expression of the three proteins having clinical features and prognosis were evaluated.

**Results:**

The expression of PABPC1 was associated with age, recurrence, and treatment but not with gender, TNM classification, or the expression of Ki-67, p53, or EBER. High expression of PABPC1 was associated with poor overall survival (OS) and disease-free survival (DFS) and was an independent predictor depending on multivariate analysis. Comparatively, no significant correlation was observed between the expression of p53, Ki-67, and EBER and survival. In this study, 120 patients received treatments and revealed significantly better OS and DFS than the untreated 37 patients. PABPC1 high expression was an independent predictor of shorter OS in the treated (HR = 4.012 (1.238–13.522), 95% CI, *p* = 0.021) and the untreated groups (HR = 5.473 (1.051–28.508), 95% CI, *p* = 0.044). However, it was not an independent predictor of shorter DFS in either the treated or the untreated groups. No significant survival difference was observed between patients with docetaxel-based induction chemotherapy (IC) + concurrent chemoradiotherapy (CCRT) and those with paclitaxel-based IC + CCRT. However, when combined with treatment and PABPC1 expression, patients with paclitaxel-added chemoradiotherapy plus PABPC1 low expression had significantly better OS than those who underwent chemoradiotherapy (*p* = 0.036).

**Conclusions:**

High expression of PABPC1 is associated with poorer OS and DFS among NPC patients. Patients with PABPC1 having low expression revealed good survival irrespective of the treatment received, indicating that PABPC1 could be a potential biomarker for triaging NPC patients.

**Supplementary Information:**

The online version contains supplementary material available at 10.1186/s12885-023-10629-4.

## Background

Nasopharyngeal carcinoma (NPC) is a malignant tumor having a unique ethnic and geographical distribution occurring in southern China and Southeast Asia [1]. Currently, new radiotherapy (RT), chemotherapy (CT), and surgical techniques are used to treat NPC, producing a satisfactory five-year survival rate among patients [2]. However, some patients still undergo tumor recurrence and metastasis, and the tumor-lymph node-metastasis (TNM) staging system for clinical prognostication and treatment decisions is insufficient to improve overall survival [3–5]. Thus, developing novel strategies using molecular markers for better diagnosis and prognosis of NPC patients is necessary.

According to the World Health Organization (WHO) classification, NPC is classified into squamous cell carcinoma (SCC), nonkeratinizing carcinoma, and undifferentiated or poorly differentiated carcinoma. Epstein-Barr virus (EBV) plays an essential aetiological role in the genesis of nonkeratinizing undifferentiated NPC [6]. The NCCN Clinical Practice Guidelines for Head and Neck Cancers (Version 2.2020) have indicated testing the EBV status in undifferentiated and nonkeratinizing NPC tumors. EBV genomes are commonly detected using polymerase chain reaction (PCR) in most NPC cells. In contrast, the two EBV-encoded small RNAs (EBER-1/2) are widely detected using in situ hybridizations (ISH) across all EBV-infected tumor types [7]. The ISH for EBERs, such as EBER1/2, is the gold standard for identifying EBV-related pathological lesions [8].

Nonkeratinizing carcinoma, whether differentiated or undifferentiated, is sensitive to CT and RT. RT single or combined chemoradiotherapy treatment is the primary regimen for NPC patients with early and locally advanced tumors [9], leading to only about 15% of patients suffering death or recurrence [2]. The biggest obstacle in treating these NPCs is reducing recurrence and improving CT efficiency. However, for recurrent and metastatic NPC, the clinical effect of RT or CT is unsatisfactory. Systemic palliative CT is added to these recurrent metastatic carcinomas after RT. However, the prognosis of the aggressive subgroup is poor, with a median survival of only 11–22 months [10]. Molecular targeted therapy and immunotherapy have been employed to compensate for the deficiency of traditional treatments and showed satisfactory efficiency, which can supplement existing therapies [11–13]. Anti-EGFR monoclonal antibodies, tyrosine kinase inhibitors (TKI), and VEGF inhibitors are the primary drugs for targeted molecular treatment of NPC, impacting many cancers combined with CT or concurrent chemoradiotherapy (CCRT) [14, 15]. However, the genetic characteristics of each patient should be screened before treatment since not all patients are suitable for a specific target. Therefore, it is necessary to analyze the molecular characteristics of specific NPC to better monitor and personalize the treatment of patients.

Currently, a series of molecular markers that facilitate diagnosis, prognosis, and status of the patients have been developed because of recent advances in NPC research. However, few markers are suitable for clinical application, despite the favorable characteristics of several developed NPC biomarkers [6]. The leading causes could be the high instability and aberrations of the NPC genome, the complexity of tumor genesis, and the heterogeneity among the individuals. Therefore, developing and applying biomarkers on different carcinogenetic pathways should help overcome these obstacles. Cytoplasmic poly(A)-binding protein 1 (PABPC1), as a significant component of cytoplasmic RNA-binding proteins, is widely distributed in the cytoplasm of eukaryotes as modulators of translation, mRNA stability, and decay, involving the mRNA post-transcriptional regulation [16, 17]. Dysregulation of PABPC1 expression is observed within multiple tumor types. PABPC1 overexpression associated with BDNF-AS overexpression inhibited the proliferation, migration, and invasion of glioblastoma cells [18]. Simultaneously, the upregulation of PABPC1 promoted malignant progression in ovarian cancer [19], hepatocellular carcinoma [20], and esophageal squamous cell carcinoma (ESCC) [21]. Therefore, the role of PABPC1 in most cancers, especially squamous cell carcinoma, is to promote malignancy. However, whether PABPC1 promotes or inhibits tumorigenesis in the EBV-associated NPC is undocumented. In this study, the expressions of PABPC1 in NPC samples were assessed, the relationship between PABPC1 expression and EBV infection was analyzed, and Ki-67 and p53 expression were investigated. Moreover, the clinical value of PABPC1 was evaluated as a prognostic biomarker for the survival of patients after treatments.

## Methods

### Patients and specimens

The histological type for NPC tumors was identified depending on the WHO 2006 classification. The TNM of NPC was defined through the American Joint Committee on Cancer (AJCC) staging system, 7^th^ Edition. The clinical and pathological information was collected, including age, gender, tumor characteristics, lymphatic invasion, and metastasis. Patients with other malignant tumors, antitumor treatment before pathologically diagnosed, or lost to follow-up were excluded. Finally, 157 formalin-fixed and paraffin-embedded (FFPE) tissues were recruited from among the NPC patients (median age 53.2 years, ranging from 15–79) from the Affiliated Hospital of Southwest Medical University between January 2014 and December 2018. All the patients were examined routinely every 3–6 months during the first five years and every 12 months in the upcoming years for follow-up. The overall survival (OS) interval from pathological diagnosis to the end of follow-up or death. The disease-free survival (DFS) interval from pathological diagnosis to end follow-up (no recurrence) or recurrence. The present study was ethically approved by the Ethics Committee of the same hospital.

### In situ hybridization (ISH)

The probe for EBER1/2 was labeled at the 3’ end using Digoxigenin. The DIG labeling EBER test kit (ISH-7001) was procured from Zhongshan Golden Bridge Bio-technology Co., Ltd., Beijing. The negative control and the procedures to achieve immunochemical staining for EBER1/2 were implemented based on the instructions. A scoring system for ISH recorded the percentage of tumor cells with nuclei staining in total tumor cells. We examined the tumor regions in the whole specimen at 100 × magnification. Low expression included 0, 1–49% (1 +), and 50–74% (2 +); high expression had 75–100% (3 +), similar to the criterion of Zeng et al. [22]. Two pathologists independently scored all the samples.

### Immunohistochemistry and evaluation

The FFPE tissue specimens were cut into 4 μm sections. These sections were dewaxed in preheated xylene and rehydrated by incubating in an ethanol gradient (100%, 95%, 90%, 80%, 70%) and then immersed in 3% hydrogen peroxide (H_2_O_2_) for 15 min. Antigen retrieval was performed through microwave in citrate buffer (pH 6.0) for PABPC1 and EDTA (pH 9.0) for Ki-67 and p53. Non-specific binding was blocked using 5% goat serum for 30 min, followed by primary antibody incubation (anti-PABPC1, Abcam, ab21060, 1:1000 dilution; anti-Ki-67 and anti-p53, Fuzhou Maixin Biotechnology Development Co., Ltd, MAB0672 and MAB0674, respectively, 1:1000 dilution) overnight at 4 °C. After washing using PBS, a goat-anti-rabbit secondary antibody was applied to the slides for 30 min at 37 °C. Then, the slides were rinsed with PBS, peroxidase substrate DAB was added for immunostaining, and counterstained with hematoxylin.

**Fig. 1:**
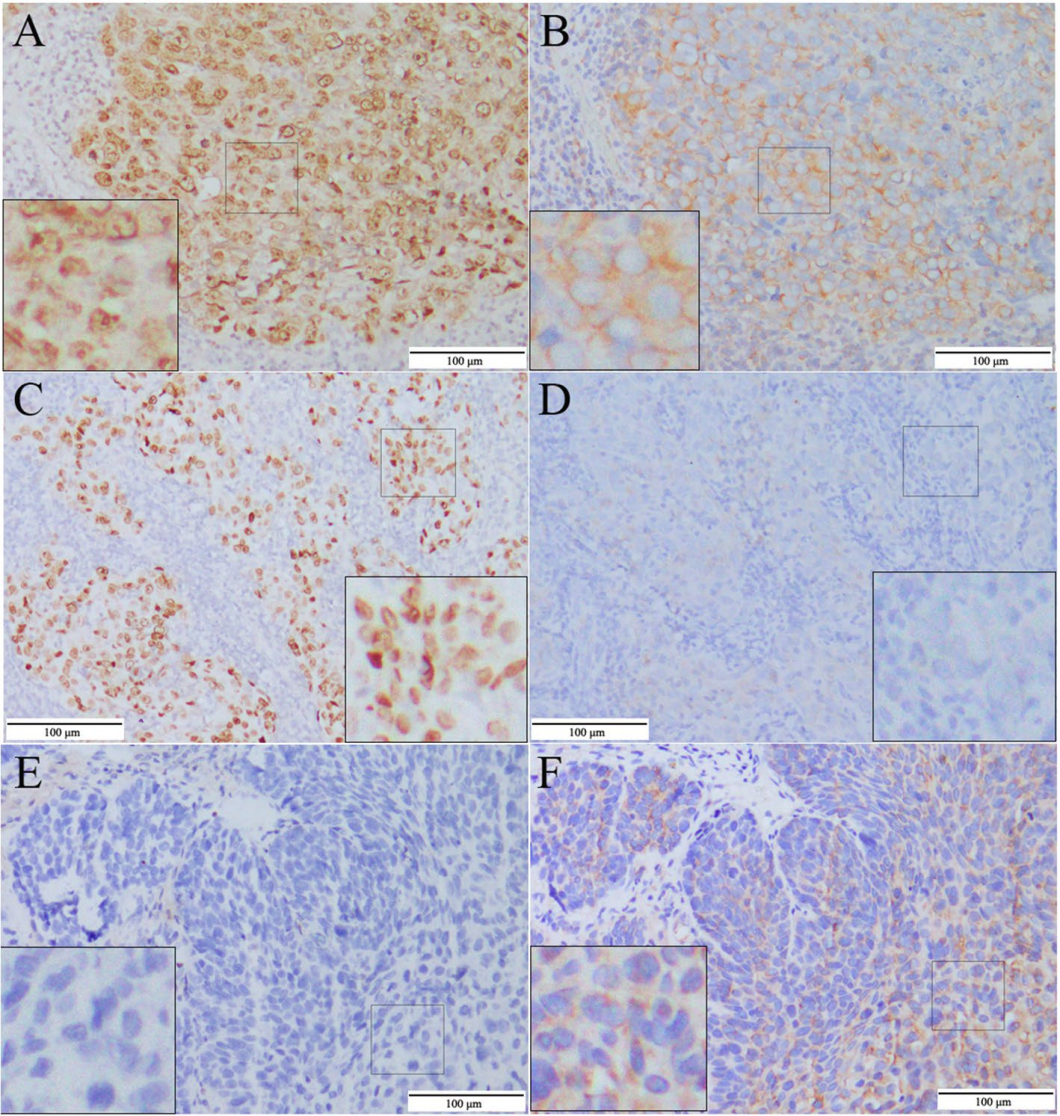
EBER1/2 and PABPC1 expression in NPC samples. **a**,**c**, and (**e**) 95%, 100%, and 0% for EBER1/2 with high expression in specimens 30 and 109 and negative expression in specimen 19, respectively; (**b**), (**d**), and (**f**) 55%, 0%, and 20% for PABPC1 having high-expression in specimen 30, low-expression in specimens 109 and 19, respectively. (Clinical and pathological characteristics of specimens 30, 109, and 19 are represented in Supplemental Table 1)

The staining was separately viewed by two pathologists blinded to the clinical or clinic-pathological status of the patients. The expression of proteins on the slides was evaluated by scanning the entire tissue specimen under low-power magnification (× 100) and then confirmed under high-power magnification (× 400). A scoring criterion depending on the percentage of positive cells in total tumor cells was used to evaluate the immunostaining of the proteins. We examined the tumor regions in the whole specimen at 100 × magnification. The high expression of PABPC1 was defined as a ≥ 25% score, and the low expression was < 25%. The cut-off value of 25% was determined based on the ROC curve (Fig. 1A). High expression of Ki-67 and p53 was defined as ≥ 50% score, and low expression was < 50%, similar to the criteria of the previous reports [23, 24].

### Treatments after pathological diagnosis

A total of 113 patients were treated with CT regimens, including concurrent chemotherapy (CC) and induction chemotherapy (IC) (Supplementary Table 1). CC has cisplatin 100 mg/m^2^, repeated every 21 days, a total of three cycles, or a weekly regimen of cisplatin 40 mg/m^2^, repeated every week. Low-toxicity alternatives, such as nedaplatin, carboplatin, lobaplatin, etc., were chosen for patients unsuitable for utilizing cisplatin. IC contains the TPF regimen [cisplatin 60 mg/m^2^, day 1; docetaxel 60–80 mg/m^2^, day 1; 5-Fu 600 mg/m^2^, day 1–5; once every three weeks], the PF regimen [cisplatin 80–100 mg/m^2^, day 1; 5-Fu 800–1000 mg/m^2^, day 1–5; once every three weeks], and the TP regimen [cisplatin 80 mg/m^2^, day 1; paclitaxel 75 mg/m^2^, day 1; once every three weeks].

In addition to 37 patients, the rest 120 received intensity-modulated RT (IMRT) for NPC treatment. Due to the low positioning error of NPC, the conventional image-guided RT was not recommended. Primary and nodal gross tumor volume (GTV) was used to screen tumors, as all the gross masses were visualized on computed tomography and/or magnetic resonance imaging. The high-risk clinical tumor volume (CTV) was the GTV plus a 5–10 mm margin. Considering the uncertain factors such as positioning error, system error, organ movement, and target area change during irradiation, PTV is recommended to expand by 3-5 mm. A total dose of *D*_T_ 68–76 Gy/30–33 fractions was applied to PTV-GTV_nx_; *D*_T_ 60–64 Gy/30–33 fractions to PTV-CTV_1_; *D*_T_ 50–54 Gy/30–33 fractions to PTV-CTV_2_; 2–2.33 Gy/fraction.

### Statistical analysis

Data were analyzed using IBM SPSS version 19.0 software (IBM, Armonk, NY, USA). The association between the expression of PABPC1 and clinic-pathological characteristics was analyzed using the chi-square test (two-tailed). Survival curves were calculated using Kaplan–Meier analysis, and the log-rank test examined differences between groups. Univariate and multivariate survival analyses were performed depending on the Cox proportional hazard model. For all the tests, *p* ≤ 0.05 was considered statistically significant.

## Results

### Clinical and histopathological characteristics of the 157 NPC cases

In the current study, 157 primary NPC patients were screened, including 114 males (53.7 ± 11.2 years, ranging from 15.0–79.0) and 43 females (51.8 ± 12.8 years, ranging from 25.0–76.0) (Supplementary Table 1). There were 71 patients ≥ 55 years and 86 patients < 55. Pearson’s correlation analysis indicated that there was no significant association between age and clinical stage (*p* = 0.218), age and smoking and drinking habits (*p* = 0.942), and tumor classification (*p* = 0.565).

### PABPC1 expression in NPC tissues

**Fig. 2:**
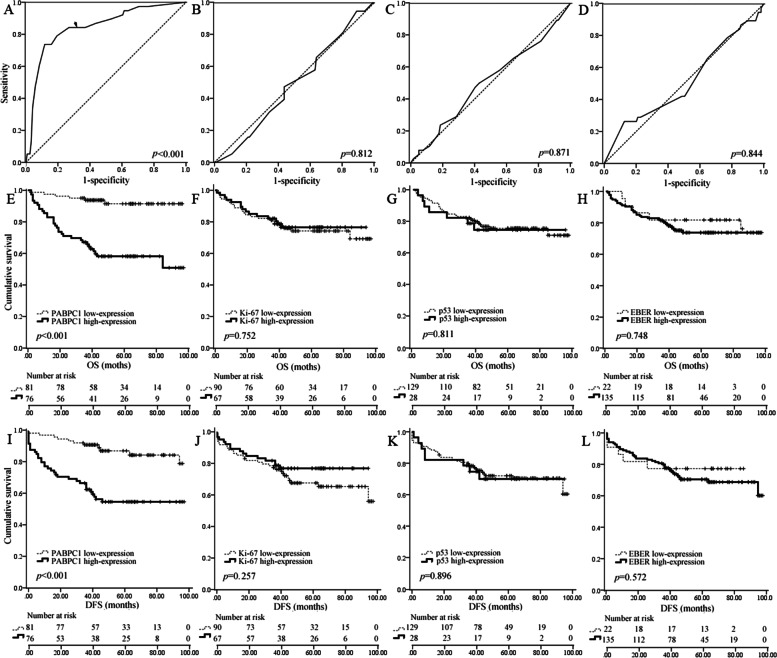
ROC curve analysis and Kaplan–Meier survival analysis. **a**-**d** ROC curve analysis of the relationship between survival and PABPC1 (**a**), Ki-67 (**b**), p53 (**c**), and EBER1/2 expression (**d**), respectively. Only PABPC1 expression was significantly related to survival; (e–h) Kaplan–Meier survival analysis of the relationship between overall survival (OS) and PABPC1 (**e**), Ki-67 (**f**), p53 (**g**), and EBER1/2 expression (**h**), respectively; (**i**-**l**) Kaplan–Meier survival analysis of the relationship between disease-free survival (DFS) and PABPC1 (**i**), Ki-67 (**j**), p53 (**k**), and EBER1/2 expression (**l**), respectively. The OS and DFS of patients differed significantly between the high-expression and low-expression groups only for PABPC1

PABPC1 protein expression was analyzed using immunohistochemistry, and staining was investigated in 128 (81.5%) samples. Unlike p53, Ki-67, or EBER staining, PABPC1 was representatively localized in the cytoplasm of the tumor region (Fig. 1). The immunohistochemistry score was recorded based on the percentage of PABPC1 expression in the tumor region. The cut-off value was 25% for PABPC1 using ROC curve analysis (Fig. 2A-D). Consequently, the 157 patients among the NPC samples were categorized into two groups: 81 and 76 patients with low (< 25%) and high (≥ 25%) PABPC1 expression, respectively. In the 81 cases with PABPC1 low expression, 52 were within 0–25% index and 29 were negative. The increased expression of PABPC1 was significantly associated with old age, tumor recurrence, and treatment but not with gender, smoking/drinking, tumor classification, TNM classification, or Ki-67, p53, and EBER expression (Table 1).

**Table 1:** Correlation between the clinicopathologic features and PABPC1 expression in 157 nasopharyngeal carcinoma patients

Characteristics	*N*	PABPC1		Statistics (χ^2^, *P* value)	EBER1/2		Statistics (χ^2^, *P* value)
		Low-expression (%)	High-expression (%)		Low-expression (%)	High-expression (%)	
Gender							
Male	114	64 (56.1)	50 (43.9)	3.447, 0.075	16 (14.0)	98 (86.0)	0.000, 0.990
Female	43	17 (39.5)	26 (60.5)		6 (14.0)	37 (86.0)	
Age							
≥ 55	71	27 (38.0)	44 (62.0)	9.549, 0.002**	6 (8.5)	65 (91.5)	3.328, 0.086
< 55 Smoking, Drinking	86	54 (62.8)	32 (37.2)		16 (18.6)	70 (81.4)	
Yes	61	32 (52.5)	29 (47.5)	0.030, 0.871	6 (9.8)	55 (90.8)	1.444, 0.229
No	96	49 (51.0)	47 (49.0)		16 (16.7)	80 (83.3)	
WHO classification							
Type III	87	45 (51.7)	42 (48.3)	1.076, 0.584	10 (11.5)	77 (88.5)	1.275, 0.529
Type II	69	36 (52.2)	33 (47.8)		12 (17.4)	57 (82.6)	
Type I	1	0 (0.0)	1 (100.0)		0 (0.0)	1 (100.0)	
Clinical stage							
I-II	36	23 (63.9)	13 (36.1)	2.828, 0.128	2 (5.6)	34 (94.4)	2.773, 0.096
III-IV	121	58 (47.9)	63 (52.1)		20 (16.5)	101 (83.5)	
T							
T1-2	89	47 (52.8)	42 (47.2)	0.122, 0.750	10 (11.2)	79 (88.8)	1.315, 0.252
T3-4	68	34 (50.0)	34 (50.0)		12 (17.6)	56 (82.4)	
N							
N0	29	15 (51.7)	14 (48.3)	0.000, 1.000	3 (10.3)	26 (89.7)	0.397, 0.529
N1-3	128	66 (51.6)	62 (48.4)		19 (14.8)	109 (85.2)	
M							
M0	153	80 (52.3)	73 (47.7)	1.162, 0.355	22 (14.4)	131 (85.6)	0.669, 0.413
M1	4	1 (25.0)	3 (75.0)		0 (0.0)	4 (100.0)	
Recurrence							
Yes	44	11 (25.0)	33 (75.0)	17.310, < 0.001**	5 (11.4)	39 (88.7)	0.356, 0.551
No	113	70 (61.9)	43 (38.1)		17 (15.0)	96 (85.0)	
Treatment							
Yes	120	68 (56.3)	52 (43.7)		19 (15.8)	101 (84.2)	1.401, 0.237
No	37	13 (36.8)	24 (63.2)	5.250, 0.025*	3 (8.1)	34 (91.9)	
Ki-67							
≥ 50%	67	36 (53.7)	31 (46.3)		7 (10.4)	60 (89.6)	1.233, 0.267
< 50%	90	45 (50.0)	45 (50.0)	0.214, 0.747	15 (16.7)	75 (83.3)	
P53							
≥ 50%	28	12 (42.9)	16 (57.1)		1 (3.6)	27 (96.4)	3.083, 0.079
< 50%	129	69 (53.5)	60 (46.5)	1.041, 0.405	21 (16.3)	108 (83.7)	
EBER							
≥ 75%	135	68 (50.4)	67 (49.6)		\	\	\
< 75%	22	13 (59.1)	9 (40.9)	0.576, 0.497	\	\	
PABPC1							
≥ 25%	76	\	\	\	9 (11.8)	67 (88.2)	0.576, 0.448
< 25%	81	\	\		13 (16.0)	68 (84.0)	

^*^P < 0 .05; **P < 0.01

### PABPC1 expression correlated with survival time

The five-year survival rate of the 157 NPC patients was 76.4%, involving 80.6% and 72.7% for stage I—II and stage III-IV subgroups, respectively. The association between PABPC1 expression and OS of NPC patients was analyzed using Kaplan–Meier analysis and log-rank test. The results indicated that NPC patients with high PABPC1 expression had significantly shorter OS and DFS time than those having low PABPC1 expression (*p* < 0.001) (Fig. 2E and I). For p53, Ki-67, and EBER, the survival time showed no significant difference between the high-expressed and low-expressed groups (Fig. 2F-H and J-L). During further analysis, patients with PABPC1 showed that high-expression did not have shorter OS and DFS time than those with low-expression at an early stage (I + II) NPC (*p* = 0.152 and *p* = 0.241, respectively). However, the patients had significantly shorter OS and DFS time at an advanced stage (III + IV) (both* p* < 0.001), depicting a correlation between PABPC1 upregulation and NPC tumor progression.

### Prognostic factors for NPC patients

In univariate analysis, older age, PABPC1 high expression, and no treatment were prognostic predictors of shorter OS and DFS in NPC patients (Table 2). In contrast, gender, TNM classification, advanced clinical stage, high-expressed p53, Ki-67, and EBER were not predictors.

**Table 2:** Univariate and multivariate analyses of prognostic factors in NPC

Variable	Univariate analysis				Multivariable analysis			
	OS		DFS		OS		DFS	
	HR (95% CI)	*P* value	HR (95% CI)	*P* value	HR (95% CI)	*P* value	HR (95% CI)	*P* value
Age, <55 vs. ≥55	5.151 (2.423-10.950)	<0.001**	2.752 (1.484-5.105)	0.001**	3.399 (1.438-8.030)	0.005**	1.703 (0.840-3.453)	0.140
Gender, male vs. female	0.637 (0.292-1.391)	0.258	0.699 (0.345-1.415)	0.319	0.340 (0.147-0.786)	0.012*	0.422 (0.198-0.902)	0.026*
T stage, T1-2 vs. T3-4	1.378 (0.729-2.606)	0.323	1.387 (0.767-2.508)	0.279	1.864 (0.867-3.017)	0.111	1.125 (0.550-2.301)	0.747
N stage, N0 vs. N1-3	1.000 (0.440-2.271)	0.999	0.739 (0.365-1.496)	0.401	0.933 (0.289-3.017)	0.908	0.391 (0.144-1.063)	0.066
Clinical stage, I-II vs. III-IV	1.652 (0.690-3.954)	0.259	1.692 (0.753-3.798)	0.203	2.653 (0.616-11.428)	0.190	7.125 (1.809-28.068)	0.005**
p53 expression, low vs. high	1.105 (0.486-2.513)	0.811	1052 (0.489-2.264)	0.897	0.978 (0.399-2.401)	0.962	1.036 (0.440-2.441)	0.935
Ki-67 expression, low vs. high	0.900 (0.469-1.730)	0.753	0.699 (0.374-1.307)	0.262	0.710 (0.338-1.491)	0.365	0.676 (0.334-1.369)	0.277
EBER expression, low vs. high	1.166 (0.455-2.992)	0.749	1.307 (0.513-3.325)	0.575	0.742 (0.268-2.054)	0.565	0.892 (0.330-2.410)	0.821
PABPC1 expression, low vs. high	6.788 (2.837-16.244)	<0.001**	3.889 (1.964-7.702)	<0.001**	5.376 (2.110-13.694)	<0.001**	3.311 (1.580-6.940)	0.002**
Treatment, no vs. yes	0.137 (0.071-0.262)	<0.001**	0.176 (0.097-0.319)	<0.001**	0.097 (0.043-0.215)	<0.001**	0.092 (0.043-0.197)	<0.001**
Drugs, Docetaxel vs. Paclitaxel	0.473 (0.153-1.463)	0.194	0.894 (0.342-2.338)	0.820	0.841 (0.228-3.099)	0.794	1.768 (0.586-5.332)	0.312
Combination, Paclitaxel + PABPC1 low-expression vs. other regimens	7.305 (0.941-56.716)	0.057	1.542 (0.546-4.351)	0.414	4.117 (0.394-43.028)	0.237	1.112 (0.231-5.350)	0.895

*NPC* Nasopharyngeal carcinoma, *HR* Hazard ratio, *CI* Confidence interval, *OS* Overall survival, *DFS* Disease-free survival

* and **, *P* value < 0.05 and 0.01 were used to determine whether differences were statistically significant between the two compared groups.

In multivariate analysis, high PABPC1 expression and no treatment were independent predictors of shorter OS and DFS. Older age was an independent predictor of the shorter OS but not DFS. However, the advanced stage (III + IV) was an independent predictor of shorter DFS. Unexpectedly, the females were an independent predictor of longer OS and DFS than males.

### Prognostic value of PABPC1 in NPC patients with a regimen

**Fig. 3:**
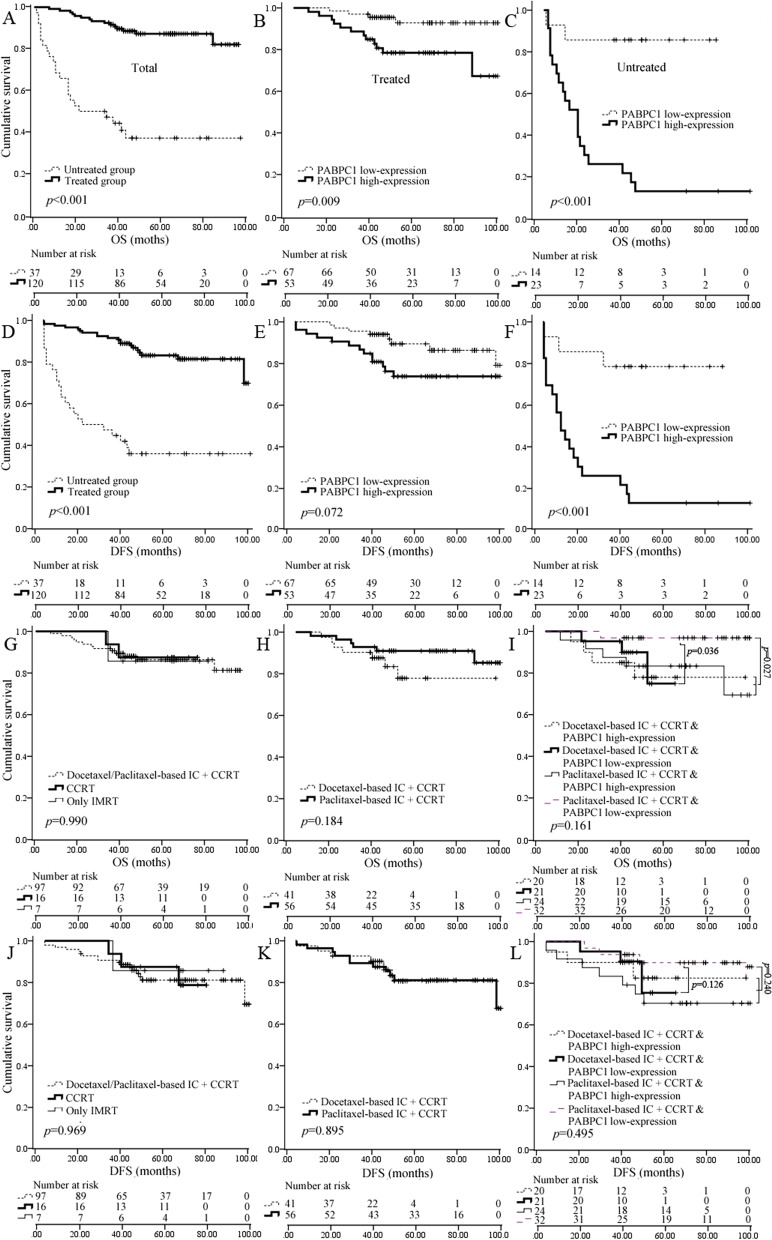
The Kaplan–Meier survival analysis according to regimens. The overall survival (OS) (**a**) and disease-free survival (DFS) (**d**) of treated patients were significantly better than those of the untreated; Patients with PABPC1 low expression had significantly better OS but not DFS than those with PABPC1 high expression in the treated group (**b** and **e**); In contrast, patients with PABPC1 low-expression had significantly better OS and DFS in the untreated group (**c** and **f**); There was no difference for OS (**g**) or DFS (**j**) among the patients with paclitaxel/docetaxel-based IC + CCRT, the patients with CCRT, and the patients with IMRT only; There was no difference for OS (**h**) or DFS (**k**) between patients with paclitaxel-based IC + CCRT and patients with docetaxel-based IC + CCRT; OS (**i**) but not DFS (**l**); Patients with paclitaxel-based IC + CCRT and PABPC1 low-expression was significantly better than those with other chemoradiotherapy regimens

Among the 157 NPC patients, seven received IMRT only, 16 received CCRT, 97 received docetaxel/paclitaxel-based IC + CCRT, and 37 received no treatment. The regimen was significantly related to longer OS and DFS time (Fig. 3A and D). Subsequently, the five-year survival rate of treated patients was much higher than untreated (88.2% vs. 40.5%, *p* < 0.001). Patients with high PABPC1 expression have a significantly shorter OS and DFS time in the untreated group, while the treated group has shorter OS but not DFS time (Fig. 3B-C and E–F). Comparatively, patients with p53, ki-67, or EBER high expression depicted no difference in OS or DFS time from those with low expression (Supplementary Fig. 1). Multivariate analysis revealed that PABPC1 high expression was an independent predictor of shorter OS in the treated (HR = 4.012 (1.238–13.522), 95% CI, *p* = 0.021) and untreated (HR = 5.473 (1.051–28.508), 95% CI, *p* = 0.044) groups. However, it was not an independent predictor of shorter DFS in either the treated (HR = 2.034 (0.816–5.067), 95% CI, *p* = 0.127) or untreated (HR = 3.620 (0.835–15.519), 95% CI, *p* = 0.086) groups.

This study compared the effects between docetaxel and paclitaxel-added regimens. The results indicated that docetaxel-induced CT patients had no significant difference between OS or DFS time and those paclitaxel-induced (Fig. 3G-H and J-K). However, patients with PABPC1 low expression and those with paclitaxel-based IC + CCRT had longer OS time than those with docetaxel-based IC + CCRT (*p* = 0.036) (Fig. 3I and L) significantly when we combined the expression of PABPC1 with treatments. Further analysis indicated that patients with paclitaxel-based IC and PABPC1 low expression had a longer OS time than the other patients with chemoradiotherapy (*p* = 0.027) significantly, indicating that PABPC1 expression combined treatment could potentially distinguish low-risk patients from the total.

## Discussion

In this study, we developed a prognostic biomarker, PABPC1, to predict the OS and DFS of NPC patients irrespective of whether or not they underwent treatment. The expression of PABPC1 was localized inside the cytoplasm of tumor cells. It could hardly investigate in normal epithelial tissues, the same as in esophageal squamous cell carcinoma (ESCC) [21], since the two carcinomas were derived from squamous cells.

NPC, especially nonkeratinizing carcinoma, is closely associated with EBV infection. Previous studies have depicted that the pretreatment plasma EBV DNA level is one of the most significant factors for the diagnosis and prognosis of NPC [25]. Unlike EBV DNA, the prognostic value of EBER1/2 in nonkeratinizing NPC remains unclear, though the overexpression of EBER1 had a good prognosis for NPC [22]. Our results demonstrated that EBER1/2 expression was not associated with OS or DFS in the treated or untreated groups. That may be because both EBER1 and EBER2 are highly expressed in latent cells. However, the latter form promotes malignant behavior and antagonizes the function of the former [26, 27].

Though the positive status of p53 was related to poor survival of NPC patients [28], our results revealed no correlation between p53 expression and OS or DFS survival. It should not be because the cut-off value for distinguishing high and low expression of p53 was 50%. The ROC curve analysis also failed to reveal a significant association between p53 expression and survival. The situation of proliferation indicator Ki-67 expression in NPC was similar to p53. Though positive staining of Ki-67 was detected in each sample, no significant association was observed between Ki-67 expression and survival. It differed from many studies, indicating a significant correlation between Ki-67 positive expression and poor survival [29]. It was similar to the results of Hui et al. [30] and Ben-Haj-Ayed et al. [31]. We estimated that EBV infection causes different genetic characteristics of tumors from people in other regions, resulting in additional regulation of biomarkers.

PABPC1 is the major isoform of poly(A) binding proteins and is essential in mRNA stabilization, degradation, and translation enhancement [16, 17]. Thus, the dysregulation of PABPC1 is associated with many cellular processes, such as tumorigenesis. Our previous study demonstrated that PABPC1 is a promoter of ESCC genesis and can be used as a prognostic biomarker to predict the survival of ESCC patients [21]. In this study, we investigated the characteristics of PABPC1 protein expression within NPC tissues. The expression pattern of PABPC1 in NPC was similar to that in ESCC could be due to the squamous derivation of tumor cells. However, the average percentage of staining tumor cells in NPC was lower than in ESCC, leading to a lower cut-off value of PABPC1 expression in NPC. We estimated that the tumor cells in that NPC were closely arranged and had large nuclei since most NPC (56.3%) were undifferentiated types, resulting in some stained cytoplasm being blocked.

RT or RT-based comprehensive treatment is the most effective curative NPC treatment. With the extensive application of RT, especially IMRT, the local control and survival rates of NPC have been significantly improved, despite some failures primarily due to distant metastasis [3, 32, 33]. The present study investigated much higher five-year OS and DFS survival rates in the IMRT-treated group than in the untreated group (87.5% vs. 40.5% for OS and 84.2% vs. 37.8% for DFS). Furthermore, most patients treated with IMRT also received IC and/or CC. No significant differences were observed in OS or DFS time between patients with CCRT and patients with IMRT only or between patients with docetaxel/paclitaxel-based IC + CCRT and patients with CCRT. It could be because the number of patients in the two groups (IMRT only and CCRT group, respectively) was too small (7 and 16, respectively). However, an excellent analysis of 2605 patients from eight studies indicated that IC + CCRT was not significantly more effective than IC + RT for managing locally advanced NPC due to more toxicity [34]. Thus, the chemotherapeutic regimen added to IMRT may not benefit patients easily unless reducing adverse factors, including NPC chemoresistance, chemotherapeutic toxicity, and complications [2].

IC was considered a less toxic and tolerable regimen for NPC patients than CC. Despite some adverse reactions, paclitaxel and docetaxel have been widely used as effective antitumor drugs in the IC regimens for NPC. The upgrade from ‘liposomal paclitaxel’ in which paclitaxel is encapsulated within liposome has also been identified as effective as docetaxel in IC regimen for NPC [35]. However, not all patients could be suitable for the paclitaxel or docetaxel regimen, whether with or without CC. Considering that EBV-caused nonkeratinizing NPC is closely related to genetic characteristics, further classifying the NPC patients into subgroups using molecular markers could be good for personalizing regimens [6, 36]. In this study, the five-year OS rate of patients with paclitaxel-based IC + CCRT was as high as that of patients with docetaxel-based IC + CCRT because it was insignificant (*p* = 0.184). However, patients with paclitaxel-based IC + CCRT in the PABPC1 low-expression group depicted significantly longer OS time than those with docetaxel-based IC + CCRT (*p* = 0.036) when we divided the patients into PABPC1 low-expression and high-expression groups. It showed that paclitaxel could be a better drug for patients with PABPC1 low expression than docetaxel. Further analysis depicted that patients with paclitaxel added regimen plus PABPC1 low-expression had significantly better OS than those who underwent chemoradiotherapy (*p* = 0.027). The five-year OS of untreated patients with PABPC1 low expression was close to that of the patients who underwent a regimen (85.7% vs. 87.5), suggesting it may not be necessary for patients with PABPC1 low expression to receive the high intensity of CT or RT. However, it is essential for patients with high PABPC1 expression.

## Conclusions

The current study depicted that PABPC1 expression is associated with NPC malignancy and may represent a novel and critical prognostic marker for NPC patients. High expression of PABPC1 predicts poor OS and DFS survival in untreated patients, with poor OS in treated patients. The combination of PABPC1 expression and regimen revealed that paclitaxel-based IC + CCRT is a better regimen for patients with PABPC1 low expression than the other regimens, suggesting that PABPC1 could potentially triage NPC patients and enhance personalized treatment.

## Supplementary Information

**Additional file 1:** **Supplementary Fig. 1.** The Kaplan-Meier survival analysis according to p53, Ki-67, EBER1/2 expression, and treatment.

**Additional file 2:** **Supplemental Table 1.** Clinical and pathological characteristics of the 157 NPC patients.

## Data Availability

All the data generated or analyzed during this study have been included in this published article [and its supplementary information files].

## Abbreviations

AJCC: American joint committee on cancer
CC: Concurrent chemotherapy
CCRT: Concurrent chemoradiotherapy
CT: Chemotherapy
CTV: Clinical tumor volume
DFS: Disease-free survival
EBER: EBV encoded small RNA
EBV: Epstein-Barr virus
ESCC: Esophageal squamous cell carcinoma
FFPE: Formalin-fixed and paraffin-embedded
GTV: Gross tumor volume
IC: Induction chemotherapy
IMRT: Intensity-modulated RT
ISH: In situ Hybridization
NPC: Nasopharyngeal carcinoma
OS: Overall survival
PCR: Polymerase chain reaction
PABPC1: Cytoplasmic poly(A)-binding protein 1
RT: Radiotherapy
SCC: Squamous cell carcinoma
TKI: Tyrosine kinase inhibitor
TNM: Tumor-lymph node-metastasis
