# Correction: Cytoplasmic poly(A)-binding protein 1 (PABPC1) is a prognostic biomarker to predict survival in nasopharyngeal carcinoma regardless of chemoradiotherapy

**DOI:** 10.1186/s12885-023-10666-z

**Published:** 2023-02-27

**Authors:** Feng Ling, Shengen Xu, Xiaochen Li, Xingwang Sun, Wenbo Long

**Affiliations:** 1 Pathology Department of the First Affiliated Hospital, Southwest Medical University, Sichuan, People’s Republic of China; 2 Department of Otorhinolaryngology-Head and Neck Surgery, the Affiliated Hospital of Southwest Medical University, Sichuan, People’s Republic of China

**Correction: BMC Cancer 23, 169 (2023)**

**https://doi.org/10.1186/s12885-023-10629-4**

Following publication of the original article [1], the authors identified an error in the author name of Feng Ling. The given name and family name were erroneously transposed.

The incorrect author name is: Ling Feng

The correct author name is: Feng Ling

The author group has been updated above and the original article [1] has been corrected.
