# Supplementary figures and images for: Cytoplasmic poly(A)-binding protein 1 (PABPC1) is a prognostic biomarker to predict survival in nasopharyngeal carcinoma regardless of chemoradiotherapy
Source: BMC Cancer. 2023 Feb 20;23:169. doi: 10.1186/s12885-023-10629-4 (PMC9940331; doi:10.1186/s12885-023-10629-4)

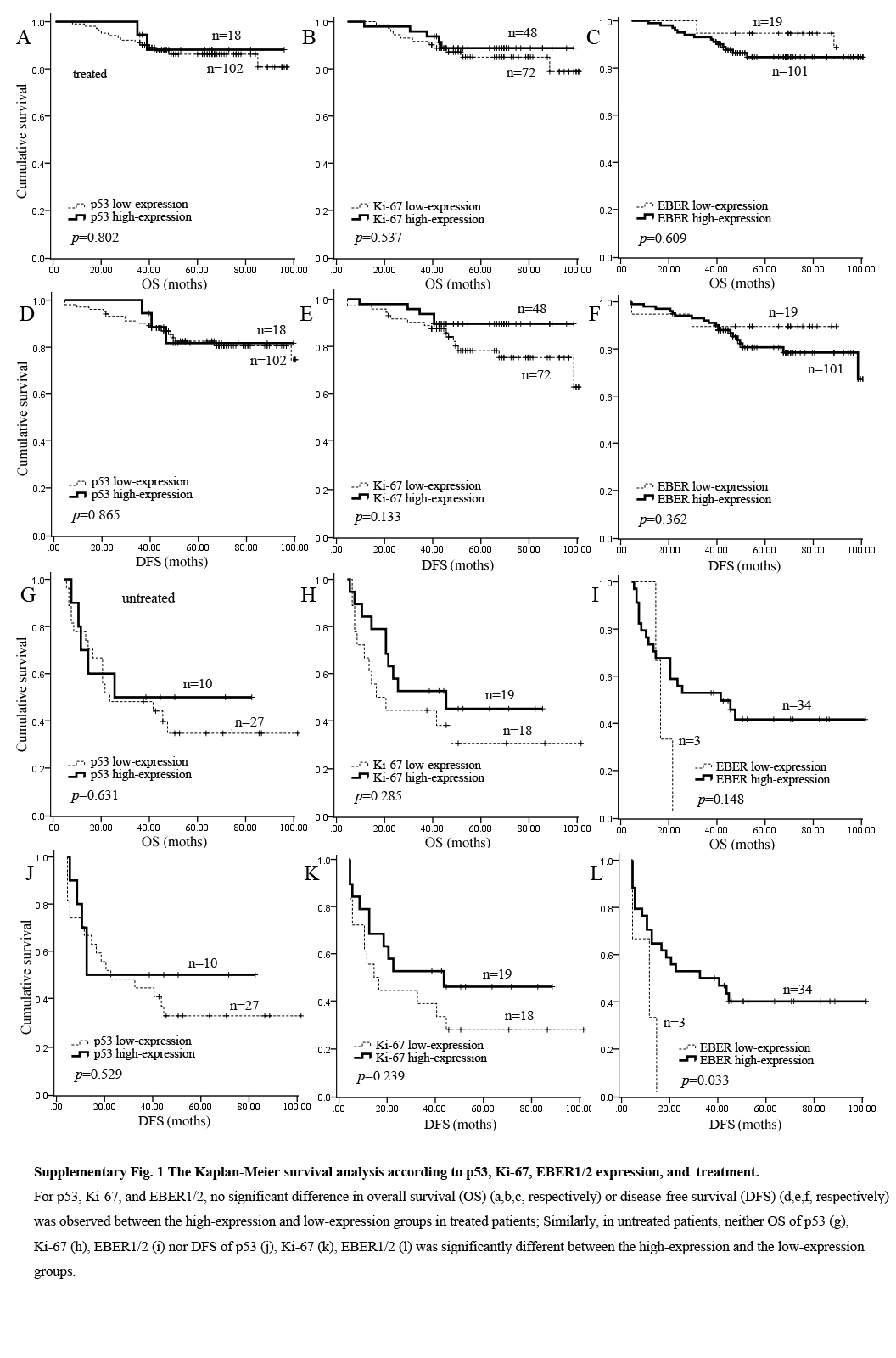

Supplement: Supplementary file 1 — Additional file 1: Supplementary Fig. 1. The Kaplan-Meier survival analysis according to p53, Ki-67, EBER1/2 expression, and treatment. [file 12885_2023_10629_MOESM1_ESM.jpg]
